# Piceatannol, a Natural Analog of Resveratrol, Exerts Anti-angiogenic Efficiencies by Blockage of Vascular Endothelial Growth Factor Binding to Its Receptor

**DOI:** 10.3390/molecules25173769

**Published:** 2020-08-19

**Authors:** Wei-Hui Hu, Diana Kun Dai, Brody Zhong-Yu Zheng, Ran Duan, Tina Ting-Xia Dong, Qi-Wei Qin, Karl Wah-Keung Tsim

**Affiliations:** 1 Joint Laboratory of Guangdong Province and Hong Kong Region on Marine Bioresource Conservation and Exploitation, College of Marine Sciences, South China Agricultural University, Guangzhou 510642, China; whuaf@connect.ust.hk (W.-H.H.); qinqw@scau.edu.cn (Q.-W.Q.); 2 Shenzhen Key Laboratory of Edible and Medicinal Bioresources, The Hong Kong University of Science and Technology, Hi-Tech Park, Nanshan, Shenzhen 518057, China; dianadk@ust.hk (D.K.D.); brodyz@ust.hk (B.Z.-Y.Z.); duanran@ust.hk (R.D.); botina@ust.hk (T.T.-X.D.); 3 Division of Life Science and State Key Laboratory of Molecular Neuroscience, The Hong Kong University of Science and Technology, Hong Kong 999077, China

**Keywords:** piceatannol, herbal medicine, vasculogenesis, VEGF, VEGFR2, pure compound

## Abstract

Piceatannol is also named as *trans*-3,4,3′,5′-tetrahydroxy-stilbene, which is a natural analog of resveratrol and a polyphenol existing in red wine, grape and sugar cane. Piceatannol has been proved to possess activities of immunomodulatory, anti-inflammatory, antiproliferative and anticancer. However, the effect of piceatannol on VEGF-mediated angiogenesis is not known. Here, the inhibitory effects of piceatannol on VEGF-induced angiogenesis were tested both in vitro and in vivo models of angiogenesis. In human umbilical vein endothelial cells (HUVECs), piceatannol markedly reduced the VEGF-induced cell proliferation, migration, invasion, as well as tube formation without affecting cell viability. Furthermore, piceatannol significantly inhibited the formation of subintestinal vessel in zebrafish embryos in vivo. In addition, we identified the underlying mechanism of piceatannol in triggering the anti-angiogenic functions. Piceatannol was proposed to bind with VEGF, thus attenuating VEGF in activating VEGF receptor and blocking VEGF-mediated downstream signaling, including expressions of phosphorylated eNOS, Erk and Akt. Furthermore, piceatannol visibly suppressed ROS formation, as triggered by VEGF. Moreover, we further determined the outcome of piceatannol binding to VEGF in cancer cells: piceatannol significantly suppressed VEGF-induced colon cancer proliferation and migration. Thus, these lines of evidence supported the conclusion that piceatannol could down regulate the VEGF-mediated angiogenic functions with no cytotoxicity via decreasing the amount of VEGF binding to its receptors, thus affecting the related downstream signaling. Piceatannol may be developed into therapeutic agents or health products to reduce the high incidence of angiogenesis-related diseases.

## 1. Introduction

It was firstly proposed in the early 1970s that the new formation of blood vessels was a prerequisite for a tumor to grow larger than a few mm^3^ in size, providing with oxygen and nutrition to support the exponential growth of tumor [1]. Since then, many investigations are focused on a possibility that inhibition of neovascularization may be helpful to prevent and delay cancer growth and to release patients suffering from cancers [2,3]. The growth of new blood vessel, also defined as angiogenesis, is a network composing of new blood vessels formed on a basis of pre-existing vessels. A large number of proteins and its mechanism involved in the regulation of this process have been identified [4,5,6]. Among these proteins related with vessel growth, vascular endothelial growth factor A (VEGF) has been shown to be the most significant angiogenic factor, secreted by tumor [7,8]. VEGF activates various cellular functions, including angiogenesis via binding and activating two mainly endothelial cell surface receptors, i.e., VEGF receptor 1 (VEGFR1, also known as FLT1) and VEGFR2 (also known as KDR and FLK-1) [9,10]. VEGFR2 is required for vascular development of endothelial cells, and the affinity value of VEGF binding to VEGFR2 is much higher than that to VEGFR1 [11,12]. After activated by VEGF, both VEGFR1 and VEGFR2 separately experience signal transduction, and VEGFR2 demonstrates stronger ligand-dependent tyrosine phosphorylation, as compared with that of VEGFR1 [13,14].

Cancer cells are extremely easy to undergo mutation, causing cancer cells to become drug tolerant and bringing an obstacle in clinical treatments of cancers [15,16]. By combining multidisciplinary and interdisciplinary features of multidrug-resistant cancers, Assaraf et al. [16] had provided evidence in deciphering mechanism underlying anticancer drug resistance and which paved the way towards development of novel modalities for precision medicine treatment. In contrast, endothelial cells, the primary components in forming blood vessels, are normal diploid cells and possess a relatively lower rate to suffer from mutation. Having this genetic stability, the possibility of anticancer treatment focusing on tumor-induced angiogenesis is proposed to be fewer in suffering from drug tolerance. Angiogenesis exists in limited circumstance in adult; while in comparison with chemotherapy for cancer treatment, the side effect of anticancer therapy targeting angiogenesis is suspected to be relatively limited [17]. This treatment may be applied to various kinds of cancers: because angiogenesis is needed during the growth and development of cancer. Bevacizumab, also called as Avastin, was approved by US Food and Drug Administration (FDA) and has been used to treat different types of cancers, including lung cancer, colon cancer, renal-cell carcinoma and glioblastoma [18]. As a recombinant humanized monoclonal antibody, Bevacizumab can be only given by injection, and it exerts effects through suppressing new blood vessel formation by binding to VEGF [19].

It is of great benefits and values for cancer treatment in terms of anti-angiogenic prevention and treatment. The natural edible compounds from traditional Chinese medicine (TCM) or food having anti-angiogenic effects could be useful for prevention of cancer progression. Piceatannol is also named 3,3′,4,5′-*trans*-tetrahydroxy-stilbene or *trans*-3,4,3′,5′-tetrahydroxy-stilbene and which is an analog of resveratrol and is a natural phenolic compound. Piceatannol is derived from many kinds of fruits, including berries, sugar cane, peanut, passion fruit seed, red wine, grape and white tea [20,21]. Piceatannol is being generated during the ripening process of grapes and in the process of fermentation triggered by β-glucosidases of microorganism [22]. Investigations have also shown that piceatannol could be produced from metabolism of resveratrol via hydroxylation with effects of cytochrome P4501B1 [23,24]. In addition to an additional hydroxyl group located at the 3′-carbon, the structure of piceatannol is identical to resveratrol, and, of note, it has been shown that piceatannol is more stable than resveratrol during metabolism process [25]. Moreover, piceatannol has been proved to possess similar effects to resveratrol [26], and it has been demonstrated that piceatannol could be applied as health products to reduce high incidence of cardiovascular diseases [27]. Moreover, piceatannol was reported to be a natural compound with therapeutic effects, including prevention of arrhythmia, hypercholesterolemia, angiogenesis and atherosclerosis, vasorelaxation, anticancer and antioxidant activities [28].

In our previous study, resveratrol was shown to exert inhibitory effects on VEGF-mediated angiogenesis [29]. However, investigations giving support to anti-angiogenic activities of piceatannol are still very few and whether piceatannol can suppress tumor-mediated angiogenesis or related signaling mechanism of such suppressive effects is still undiscovered. Here, we have determined whether piceatannol could exert tumor suppressing effects via suppression of angiogenesis based on both cell and animal model. We also arose the possibilities of piceatannol exerting anti-angiogenic function via VEGF signaling.

## 2. Results

### 2.1. Piceatannol Suppresses VEGF-Mediated Angiogenesis

**Figure 1:**
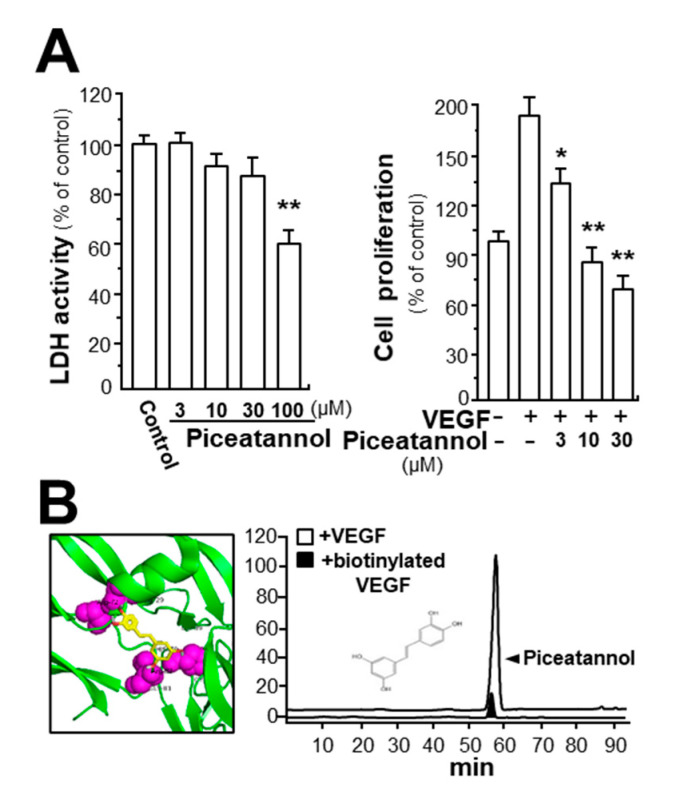
Piceatannol suppresses vascular endothelial growth factor A (VEGF)-induced cell proliferation and binding with VEGF. (**A**) Cultured endothelial cells (HUVECs) were incubated with a series of concentrations of piceatannol for 48 h and cytotoxicity detection kit plus (LDH) (left panel) and 3-(4,5-dimethylthiazol-2-yl)-2,5-diphenyltetrazolium bromide (MTT) (right panel) assays were performed. HUVECs, seeded into each well of a 96-well plate with cell density set at 5000 cells/well, were treated with piceatannol for 48 h in the presence or absence of VEGF (10 ng/mL). Data are in percentage of control group, as mean ± SEM, where *n* = 4; *p* < 0.05 (*); *p* < 0.01 (**) vs. VEGF-treated group; (**B**) Chemical structure of piceatannol and protein structure, for performing molecular auto-docking, were downloaded from NCBI-PubChem database and PDB, respectively. The interaction of piceatannol binding to VEGF was demonstrated. VEGF: green; piceatannol: sticks, color of carbon: yellow, oxygen: red, hydrogen: silver; the proposed binding site: purple (right panel). UPLC chromatogram was applied to determine the amount of piceatannol in supernatant after biotinylated VEGF or VEGF (100 ng/mL) in an immunoprecipitation assay by streptavidin magnetic beads, *n* = 3 (right panel).

Piceatannol is an analog of resveratrol commonly found in fruits. Effects of piceatannol on proliferation of cultured endothelial cells (HUVECs) were determined. First, piceatannol treatment in endothelial cells exerted no effects on cell viability up to 30 µM, and this concentration was used thereafter (Figure 1A, left panel). However, the VEGF-induced endothelial cell proliferation was significantly suppressed after application of piceatannol, and the inhibitory rate was shown in a dose-dependent manner (Figure 1A, right panel). At the highest concentration of 30 µM, piceatannol had suppressive rate of over 100% in comparison with VEGF-induced group. Thus, piceatannol having no toxicity on cell viability could remarkably attenuate the VEGF-triggered cell proliferation in cultured endothelial cells. In the VEGF-treated HUVECs, VEGF application potentiated cell proliferation in a concentration-dependent manner, and the maximum change was at a concentration of 10 ng/mL (Supplementary Figure S1). The EC_50_ of piceatannol-inhibited cell proliferation was revealed at 5.11 μM (Supplementary Figure S2A). Application of piceatannol alone in cultured endothelial cells exhibited no effect on cell proliferation, cell migration, cell invasion and tube formation, i.e., the target of piceatannol should be VEGF instead of cell receptors (Supplementary Figure S2B).

In order to figure out molecular mechanism responsible for anti-angiogenic function of piceatannol, we first, determined the possible binding of piceatannol on VEGF. With application of molecular docking analysis, the potential binding activity between piceatannol and VEGF protein was demonstrated (Figure 1B). By using vina software, the affinity value representing the binding of piceatannol to VEGF protein was −7.5 to −7.1. The specific binding site of piceatannol to VEGF protein was proposed to be located at its receptor 2 binding region. In addition, the binding interaction of piceatannol to VEGF in vitro was further confirmed by performing an immuno-precipitation assay here. The amount of piceatannol in the supernatant, treated by VEGF, was much higher than that in the group incubated with biotinylated VEGF (Figure 1B).

We explored the possible binding activities among piceatannol analogs, e.g., pinosylvin and 3,4′,5-trimethoxy-transstilbene, with VEGF protein. The possible binding affinity values of pinosylvin (−6.0–−5.3) and 3,4′,5-trimethoxy-transstilbene (−6.1–−5.6) to VEGF were both lower than that of piceatannol and VEGF, representing pinosylvin and 3,4′,5-trimethoxy-transstilbene could have weaker binding with VEGF than piceatannol (Supplementary Figure S3A). As expected, pinosylvin and 3,4′,5-trimethoxy-transstilbene could suppress the VEGF-mediated cell proliferation in endothelial cells. However, the suppression was weaker than that of piceatannol (Supplementary Figure S3B).

**Figure 2:**
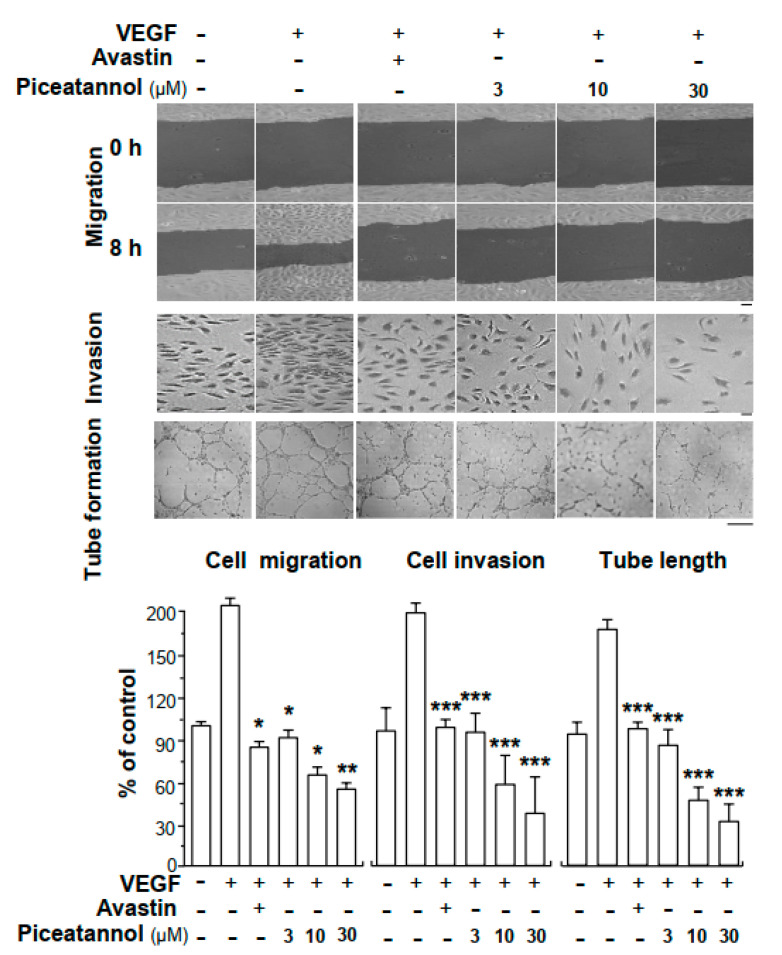
Piceatannol inhibits VEGF-mediated endothelial cell migration, cell invasion and tube formation. In cell migration assay, HUEVCs, at a density of 2 × 10^5^/well were seeded into each well of a sterile 12-well plate. A wound located in the middle of cell monolayer was made manually. Pictures representing wounds were taken separately at 0 h and 8 h by using a phase-contrast microscope. Cells were treated with VEGF in the presence or absence of piceatannol. In cell invasion assay, 100 μL of cell cultures suspended in fresh medium was plated onto the upper part of chamber of a piece of a 12-well Transwell plate. In the upper compartment, cells were treated by VEGF with or without piceatannol. In the lower chambers, 500 μL fresh medium was applied. Drug application lasted for 24 h, and cells invaded into the lower chambers were analyzed and quantified by counting cell numbers manually after fixation. In tube formation assay, each well of a 24-well plate was precoated with Matrigel and 1 × 10^5^ endothelial cells were seeded. Cell suspension was incubated with VEGF with or without piceatannol, and the drug treatment lasted for 8 h. Images representing cell tube-like structures were photographed under a phase-contrast microscope. To perform the quantification of pictures, three fields in one picture were randomly determined and formed branching points were analyzed and counted manually. In all cases, VEGF was used at 10 ng/mL. Avastin (200 μg/mL) served as a positive control. Data expressed as mean ± SEM of the percentage of control group, where *n* = 3; *p* < 0.05 (*); *p* < 0.01 (**); *p* < 0.001 (***) vs. VEGF-treated group. Bar = 40 μm.

In cultured HUVECs, VEGF application potentiated cell migration and invasion (Figure 2). Avastin, an inhibitor on angiogenesis in clinical application, was used as a positive control [30], which suppressed the migration, invasion and tube formation of cultured HUVECs. The effect of piceatannol in suppressing cell migration and invasion was investigated. Piceatannol attenuated the migratory and invasive abilities of endothelial cells in dose-dependent manners (Figure 2). Compared to VEGF-induced group, piceatannol at a concentration of 30 µM decreased VEGF-triggered cell migration and invasion by ~150 and ~170%, respectively. To further confirm the inhibitory activities of piceatannol on VEGF-triggered cell mobility, tube formation assay was performed in cultured endothelial cells. After application of VEGF, compared with control group, capillary-like tube was remarkably elongated (Figure 2). However, different concentrations of piceatannol treatment visibly interrupted the VEGF-strengthened capillary-like tubes. In comparison with Avastin-treated group, piceatannol exhibited better inhibitory activities at high doses.

**Figure 3:**
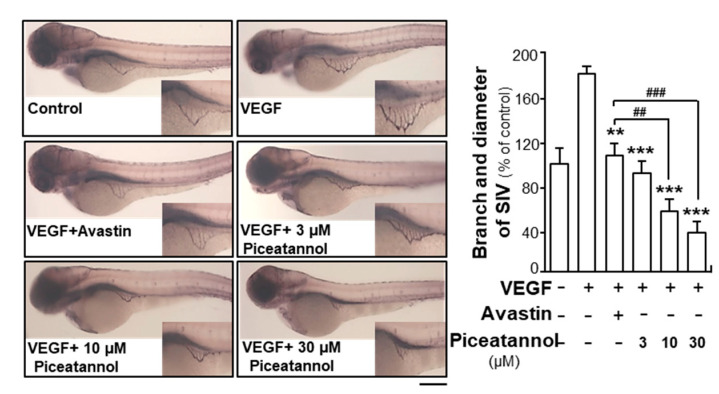
Piceatannol suppresses angiogenesis in vivo. Healthy zebrafish embryos were selected and divided into groups randomly. On 1st day of development, embryos were incubated with phenylthiourea (PTU) water containing VEGF (10 ng/mL) in the presence or absence of piceatannol at different concentrations. Avastin (200 μg/mL) served as a positive control. For the negative control, immunoglobulin prepared at the same concentration as Avastin was used here. Drug treatment lasted for 48 h and on the 3rd day development of zebrafish embryos, fish embryo staining was performed. Pictures demonstrating the subintestinal vessels and formation of blood vessels were captured. The branches and area of subintestinal vessels in different groups of drugs at different concentrations were analyzed and quantified by applying Image J software. Data expressed as mean ± SEM of the percentage of control group, where *n* = 3; *p* < 0.01 (**); *p* < 0.001 (***) vs. VEGF-treated group. *p* < 0.01 (##); *p* < 0.001 (###) vs. Avastin-treated group, as shown. Bar = 40 μm.

To investigate the effects of piceatannol on in vivo angiogenesis, we performed the study based on zebrafish embryos. The fish embryos were incubated with VEGF or Avastin or a series of concentrations of piceatannol. The application of VEGF (10 ng/mL) remarkably elongated the vessels located in the subintestinal and broadened vessel area (Figure 3). However, the co-application of VEGF and Avastin or piceatannol inhibited the vessel formation by decreasing the area of subintestinal vessels and the number of vessel branches. Piceatannol treatment exerted suppressive effect in a dose-dependent manner, and 30-µM piceatannol attenuated VEGF-mediated subintestinal vessel formation by ~140% (Figure 3). As demonstrated, the effectiveness of piceatannol at high concentration was significantly better than that of Avastin. These results suggested a key suppressive function of piceatannol in VEGF-mediated angiogenesis in vivo.

### 2.2. Piceatannol Inhibits VEGF-Mediated Signaling

**Figure 4:**
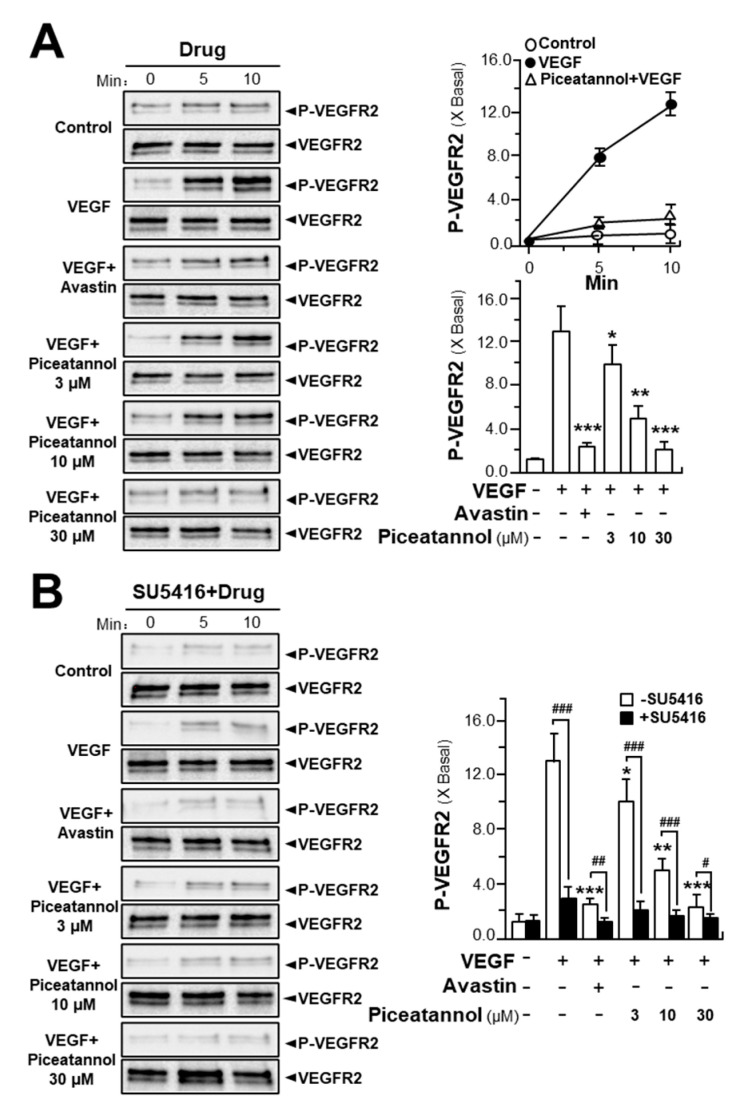
Piceatannol attenuates VEGF-induced VEGFR2 phosphorylation. (**A**) Endothelial cells were seeded into each well of a 12-well plate with cell density set at 2 × 10^5^ per well and cells were treated by VEGF (10 ng/mL) with or without piceatannol; (**B**) HUVECs were plated into each well of a 12-well plate with the density set at 2 × 10^5^ cells per well. The cells were treated with VEGF (10 ng/mL) with or without piceatannol. An inhibitor for VEGFR2 (SU5416 at 50 μM) was used. The treatment without SU5416, serving as control, was from values in Figure 4A. After the treatment, cell lysates were collected at different time points. Expressions of phosphorylated and total VEGFR2 proteins at ~210 kDa and ~230 kDa were determined by western blotting analysis. Avastin (200 μg/mL) served as a positive control. Data shown as X Basal, where the control group was set as 1, mean ± SEM, where *n* = 3; *p* < 0.05 (*); *p* < 0.01 (**); *p* < 0.001 (***) vs. VEGF-treated group; *p* < 0.05 (#); *p* < 0.01 (##); *p* < 0.001 (###) vs. its corresponding control, as shown.

**Figure 5:**
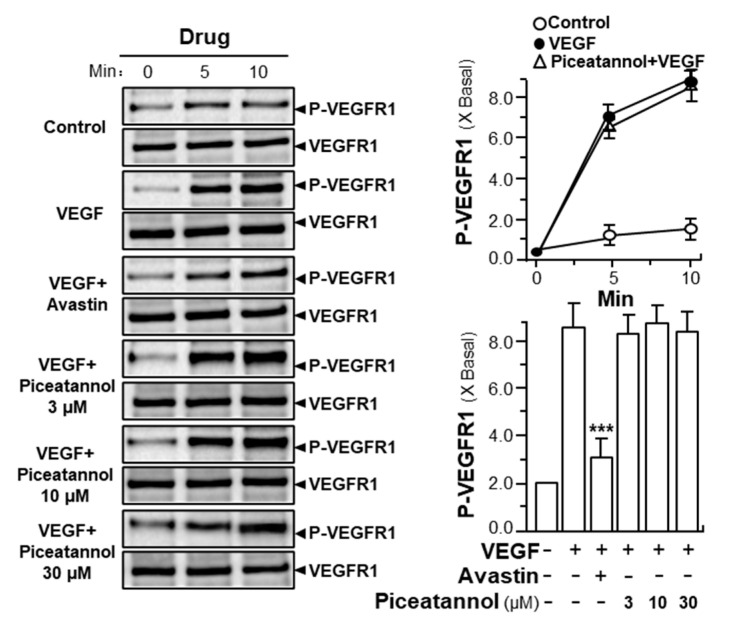
Piceatannol exerts no effects on VEGF-induced VEGFR1 phosphorylation. About 2 × 10^5^ endothelial cells were seeded into each well of a sterile 12-well plate. VEGF, at a concentration of 10 ng/mL, was used to treat cells in the presence or absence of piceatannol. After drug treatment, cell lysates were collected from each well. The phosphorylated VEGFR1 at ~180 kDa was analyzed by western blotting analysis. Avastin (200 μg/mL) served as a positive control. Results are expressed as the fold of change than control (X Basal), where the control group (no drug) was set as 1, mean ± SEM, where *n* = 3; *p* < 0.001 (***) vs. VEGF-treated group.

VEGF induces its angiogenic activity by interacting with its receptors, VEGFR1 and VEGFR2, leading to the phosphorylation of receptors and a series of downstream signaling molecules [31]. To demonstrate the effect of piceatannol in enhancing VEGF-triggered mechanism signaling in endothelial cells, the expressions of phosphorylated VEGFR1 and VEGFR2 were first, determined here. VEGFR1 and VEGFR2, as two tyrosine kinases, have been identified as VEGF receptors with high affinity values [32]. After VEGF application in HUVECs, the phosphorylation of VEGFRs was identified by western blotting test. VEGF prominently induced the activation of phosphorylated VEGFR1 and VEGFR2: both were in time-dependent manners, and the maximal activations of VEGFR1 and VEGFR2 were at ~10-fold and ~13-fold after 10 min of VEGF induction without altering the amounts of total VEGFR1 and VEGFR2 proteins (Figure 4 and Figure 5; Supplementary Figures S5 and S6). Meanwhile, we found that piceatannol application strongly suppressed VEGF-induced VEGFR2 phosphorylation in time- and dose-dependent manners in response to VEGF (Figure 4A, Supplementary Figure S5); but which exerted few effects on the phosphorylation of VEGFR1, as stimulated by VEGF (Figure 5, Supplementary Figure S6), giving support to the notion that the binding sites of piceatannol to VEGF may be located at the binding site of VEGF with VEGFR2, but not at that of VEGFR1. The maximal inhibition of piceatannol on VEGFR2 phosphorylation was at ~2-fold at 30 μM (Figure 4A, Supplementary Figure S5). To further confirm the role of piceatannol-VEGF complex in affecting its binding to VEGFR2, a VEGFR2 phosphorylation inhibitor, SU5416, was used here in cultured endothelial cells [32]. The phosphorylation, triggered by VEGF or piceatannol-VEGF complex, was fully abolished by applied SU5416 (Figure 4B, Supplementary Figure S5).

**Figure 6:**
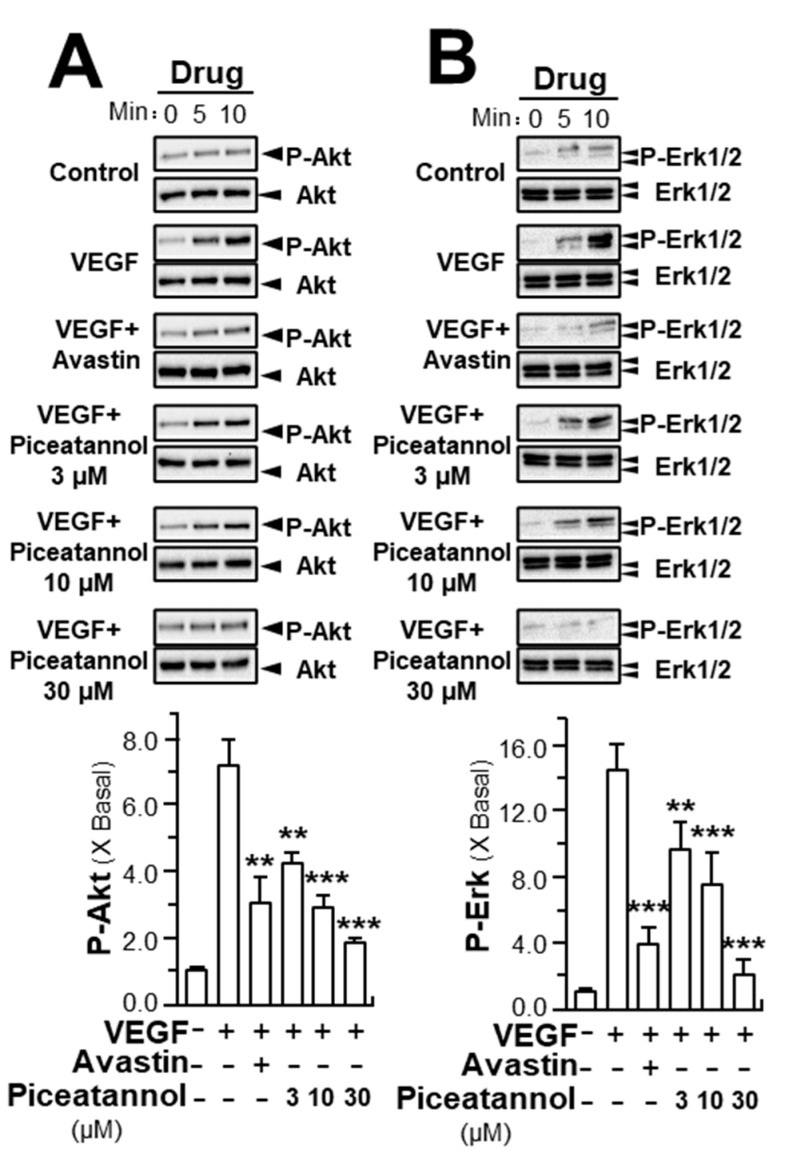
Piceatannol inhibits VEGF-induced phosphorylation of Akt and Erk. HUVECs were plated into each well of a 12-well plate at a cell density of 2 × 10^5^ cells per well. The cells were treated by VEGF (10 ng/mL) with or without piceatannol. Cell lysates were collected after drug treatment at different time points as shown. Phosphorylated and total proteins of (**A**) ~60 kDa Akt, (**B**) Erk at ~42 kDa and ~44 kDa, were separately determined by western blotting analysis. Avastin (200 μg/mL) served as a positive control. Data expressed as X Basal, where the control was set as 1, mean ± SEM, where *n* = 3; *p* < 0.01 (**); *p* < 0.001 (***) vs. VEGF-treated group.

In parallel, the expressions of key downstream molecules, triggered by VEGFR2, were further determined in HUVEC cultures after the treatment of piceatannol. As demonstrated, VEGF treatment enhanced the expressions of phosphorylated Akt and Erk by ~6 and ~14-fold; while the total proteins of Akt and Erk were unaltered (Figure 6, Supplementary Figure S7). Piceatannol on VEGF-triggered activations of Akt and Erk were identified. Obviously, decreases in phosphorylation of Akt and Erk were observed in piceatannol-treated group in time- and concentration-dependent manners. Piceatannol at 30 μM decreased the expressions of phosphorylated Akt and Erk by ~5 and ~12-fold, respectively. Avastin, a positive control, demonstrated similar suppressive activities in response to the VEGF-mediated phosphorylation of targeted molecules (Figure 6, Supplementary Figure S7).

**Figure 7:**
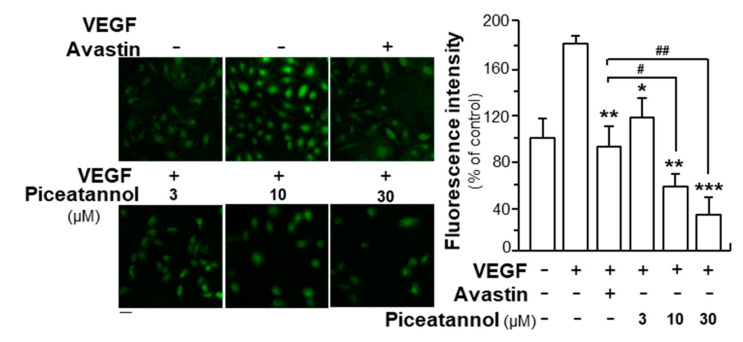
Piceatannol attenuates VEGF-triggered reactive oxygen species (ROS) formation. HUVECs with cell density set at 2 × 10^5^ cells per well were seeded into each well of a 12-well plate. After 24 h, cells were treated with VEGF (10 ng/mL) in the presence or absence of piceatannol for 48 h. After the treatment, cells were incubated with DCFH-DA at 37 °C for 30 min and the level of intracellular ROS was further analyzed by laser confocal fluorescent microscopy. Avastin (200 μg/mL) served as a positive control. Data expressed as mean ± SEM of the percentage of change in comparison to control group, where *n* = 4; *p* < 0.05 (*); *p* < 0.01 (**); *p* < 0.001 (***) vs. VEGF-treated group. *p* < 0.05 (#); *p* < 0.01 (##) vs. Avastin-treated group, as shown. Bar = 40 μm.

The formation of reactive oxygen species (ROS) is a key downstream molecule in responding to activation of VEGFR2 [33]. To further confirm the anti-angiogenic functions of piceatannol in VEGF-treated endothelial cells, ROS formation was investigated by application of DCF-DA probes. By comparing to control group, VEGF application increased ROS level by ~90%: this induction was visibly attenuated by piceatannol application, and the inhibitory rate was in a concentration-dependent manner (Figure 7). The highest suppressive rate was up to ~150%. Avastin, served as a positive control, decreased the level of VEGF-induced ROS formation by ~100%. Again, the effect of piceatannol at high concentration was significantly better than that of Avastin (Figure 7).

### 2.3. Piceatannol Suppresses VEGF-Triggered Colon Cancer Cell Proliferation and Migration

**Figure 8:**
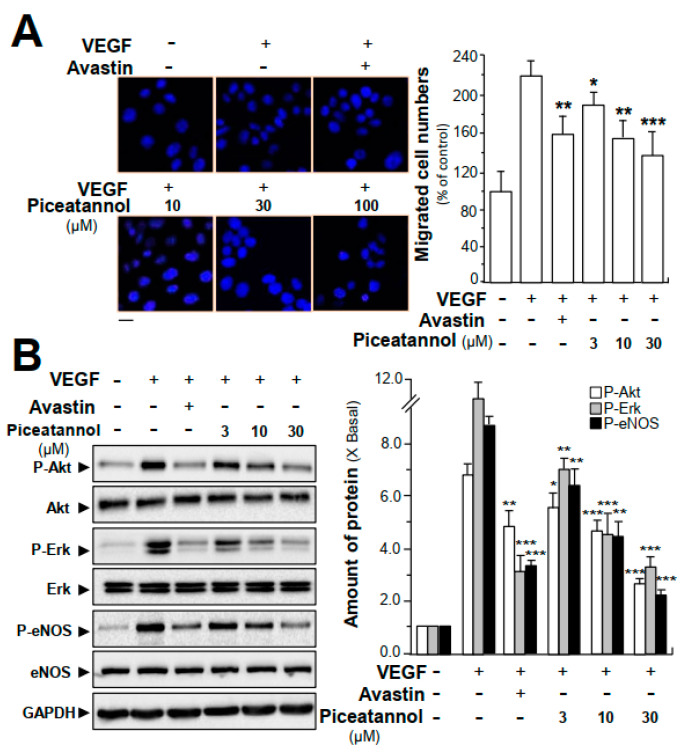
Piceatannol modulates VEGF-mediated function in colon cancer cells. (**A**) HT-29 colon cancer cells were treated with VEGF (10 ng/mL) with or without piceatannol or Avastin (200 μg/mL) for 24 h. Transwell^®^ motility assay was performed. The migrated cancer cells were counted. One of the representative images of the migrated cells is shown. The quantification data are demonstrated in right panel. Bar = 20 μm; (**B**) HT-29 colon cancer cells were treated with VEGF (10 ng/mL) in the presence or absence of piceatannol for 24 h. Total protein was collected and revealed by western blotting analysis by specific antibodies. GAPDH was used as an internal control. Quantitation (right panel) was done from the band intensity in western blotting (left panel). Avastin (200 μg/mL) served as a positive control. Data expressed as the percentage of control or the fold of change than control (X Basal), where the control group (no drug) was set as 1, mean ± SEM, where *n* = 3; *p* < 0.05 (*); *p* < 0.01 (**); *p* < 0.001 (***) vs. VEGF-treated group.

In addition to investigations on inhibitory effects of piceatannol on VEGF-induced angiogenic functions in endothelial cells, we further determined the outcome of piceatannol binding to VEGF in cancer cells. Here, we determined the anti-invasive activities of piceatannol in VEGF-treated colon cancer cells (HT-29). The results demonstrated that cells exposed to VEGF (10 ng/mL) resulted in ~2.3-fold and ~2.2-fold of increase separately in the cell proliferation (Supplementary Figure S4) and in the number of invasive cells (Figure 8A). However, the VEGF-induced cell proliferation and invasive ability was visibly reduced by piceatannol treatment in a concentration-dependent manner. Compared to VEGF-treated group, piceatannol, at a concentration of 30 μM, reduced cell proliferation and the number of invasive cells by ~120% (Supplementary Figure S4) and ~80% (Figure 8A), respectively, in cultured HT-29 cells.

Moreover, the possible roles of piceatannol in VEGF-triggered expressions of key phosphorylated molecules in cancer cells, e.g., Akt, Erk and eNOS, were further investigated by performing western blotting analysis. In comparison to control group, VEGF application, at a concentration of 10 ng/mL, in cultured cells, visibly potentiated the phosphorylation of Akt, Erk and eNOS, and the potentiations were ~7-fold, ~11-fold and ~10-fold, respectively (Figure 8B, Supplementary Figure S8). Compared with the VEGF-treated group, piceatannol was remarkably in attenuating VEGF-induced expressions of phosphorylated Akt, Erk and eNOS in dose-dependent manners, having maximal suppressive effects on phosphorylation of Akt, Erk and eNOS down to ~3-fold, ~4-fold and ~3-fold, respectively, at 30 μM (Figure 8B, Supplementary Figure S8). The amount of total Akt, Erk and eNOS were not altered in all scenarios.

## 3. Discussion

Resveratrol has been well studied in prevention and treatment of various kinds of metabolic disorders [34], and in our previous study, we have reported the anti-angiogenic activities of resveratrol [29]. However, relatively low oral bioavailability is a major barrier of resveratrol for human consumption: resveratrol is metabolized extensively, and its oral bioavailability has been estimated to be less than 1.0% [35]. As an alternative, identification of natural phytochemicals structural related to resveratrol and having improved bioavailability is of great benefits. Piceatannol, an analog and metabolite of resveratrol, has oral bioavailability of more than 1%, and this is more metabolically stable than resveratrol. Similar to resveratrol, piceatannol has been reported to possess anti-inflammatory, anticancer and cardioprotective properties [25,36]. In different types of cancer cells, applied piceatannol could induce apoptosis and suppress cell cycle progression [37,38]. In line to this notion, piceatannol was proposed to act as an estrogen receptor α agonist in human breast cancer cells [39]. Here, we hypothesized that piceatannol exerted inhibitory effects on VEGF-mediated angiogenesis through similar mechanism signaling to resveratrol; but piceatannol should be better in oral intake as health food or drug. The angiogenic activities of piceatannol were proved in different models. As the first line of evidence, piceatannol at nontoxic concentrations could significantly inhibit VEGF-mediated endothelial cell mobilities, including cell proliferation, migration, invasion as well as tube formation. Moreover, the inhibitory effects of piceatannol exerting in the zebrafish embryo subintestinal vessel formation. In terms of similar structures of piceatannol to resveratrol, the suppressive effects of piceatannol may be accounted by its binding to VEGF, attenuating the activation of VEGF to its receptor. The current results were consistent with previous studies, further proving the antiangiogenic properties of piceatannol [40,41].

Multiple processes are involved in carcinogenesis, including rapid proliferation, invasion into extracellular matrix, metastatic foci formation in different kinds of distant tissues [42]. Since early 1990s, anticancer drugs being used in clinic are targeting inhibition on cell death [43,44]. However, anticancer drugs with cytotoxic and cytostatic effects are in limited application: because which cause damage simultaneously to normal cells, resulting in serious side effects, e.g., hair loss, myelosuppression, nausea and gastrointestinal dysfunction [45]. Development of cancer cell is closely corelated with the microenvironment [46]. Blood vessels supply necessary nutrients and oxygen to the tumor, and which therefore plays a key role in metastatic cancer formation by serving as a channel for cancer cell migration. When angiogenesis is attenuated, cancer cells are unable to develop into measurable size, and thus the metastatic potential is minimized. Here, we propose application of piceatannol could weakened the VEGF-mediated cell migratory ability without toxic effects on cell viability, which suggested that piceatannol has the potentials to be developed into natural drugs for clinical anticancer treatment.

Different approaches in preclinical and clinical applications focusing on tumor microenvironments have been conducted in a wide type of cancers. For example, cetuximab and bevacizumab, existing effects in impairing tumor vasculature by blocking EGFR and VEGF, respectively, have been approved by the US Food and Drug Administration (FDA) and commercially applied in treatment of metastatic cancers [47,48]. On the other hand, age-related macular degeneration (AMD), one kind of vascular eye-related diseases, may result in the loss of vison—and based on its binding interactions with VEGF-ranibizumab has been used for AMD treatment clinically [49]. These newly developed anti-angiogenetic drugs are antibodies. Here, piceatannol, as a natural compound, could be considered as alternative in clinical cancer treatment.

Among growth factors, VEGF is a most effective inducer for angiogenesis. The expressions of VEGF and its receptors could be upregulated in tumor [7]. VEGF, secreted by tumor cells, is considered to play roles in endothelial cells via a paracrine way in forming blood vessels. VEGF exerts pro-angiogenic effects through activating its two receptors: VEGFR1 and VEGFR2. Once VEGFRs activated by VEGF, the receptors undergo conformational changes and then dimerization and auto-phosphorylation of tyrosine residues occur [11]. The binding affinity of VEGF to VEGFR1 is approximately 50-fold higher than that of VEGF and VEGFR2; however, VEGFR2 activation is mainly responsible for VEGF-triggered angiogenic activities—and which plays a vital role in VEGF-mediated downstream activities—e.g., cell survival, proliferation and migration [50]. Therefore, we targeted at the binding interactions of VEGF and VEGFR2 and analyzed the effects of piceatannol on VEGF-mediated VEGFR1 phosphorylation. Piceatannol exerted inhibitory effects on VEGFR2 phosphorylation, activated by VEGF, with no effect on total VEGFR2 protein expression. Moreover, piceatannol did not affect the VEGF-potentiated VEGFR1 phosphorylation, giving support to the specific binding site of piceatannol to VEGF at the VEGFR2 binding region. The reduced level of VEGFR2 phosphorylation may give support to the fact that expressions of molecules, including Akt, Erk and eNOS, closely belonging to VEGF–VEGFR2 signaling mechanism, were remarkably suppressed by piceatannol application in both endothelial cells and cancer cells. The inhibitory activity of piceatannol in VEGFR2 phosphorylation was not absolutely completed, as those in the VEGF-induced cell functions. Thus, we speculated that the piceatannol binding with VEGF may be unable to suppress completely VEGF-induced VEGFR activation, i.e., the VEGF-piceatannol complex could still induce VEGFR. Taken together, these molecular investigations supported the conclusion that the suppressive activities of piceatannol exerting in VEGF-induced angiogenic functions were attributed to attenuate the activation of VEGF/VEGFR2 downstream signaling pathways.

The VEGFR family is a key group of growth factor receptors responsible for angiogenesis and two receptor tyrosine kinases, VEGFR1 [51] and VEGFR2 [52], have been identified as high affinity VEGF receptors. The two receptors have common and specific ligands, characterizing by seven immunoglobulin (Ig)-like domains in extracellular region and a split kinase intracellular domain [51,52]. The receptor-binding determinants of VEGF are proposed to be localized in the amino-terminal portion (amino acids 1–110), and VEGFR1 and VEGFR2 bind with VEGF at different sites [53]. Based on the model proposed by Keyt et al. [53], the residues of VEGF, Arg82, Lys84 and His86, are involved in binding to VEGFR2; while VEGF interacting with VEGFR1 is primarily mediated by Asp63, Glu64 and Glu67. Despite their differential binding sites with VEGF, both VEGFR-1 and VEGFR-2 are essential for normal angiogenic development. In our investigation, docking analysis demonstrated that piceatannol interacted with VEGF at Arg82 residue. In addition, piceatannol application attenuated expression of VEGF-induced phosphorylated VEGFR2 with exerting no effect on VEGF-mediated VEGFR1 phosphorylation. The binding site of piceatannol to VEGF was further shown to be located at the residue of VEGF in activating VEGFR2.

## 4. Materials and Methods

### 4.1. Cell Lines and Chemicals

Human umbilical vein endothelial cells (HUVECs) were from Lonza (San Diego, CA, USA) and were cultured as previously described [50]. HT-29 human colon carcinoma cells were a gift from Prof. Randy Poon of HKUST. Cells were grown in RPMI 1640 medium supplemented with 10% heat inactivated fetal bovine serum (FBS), 1% penicillin-streptomycin and 1% *L*-glutamine. HUVECs and HT-29 cells were grown in a monolayer culture using tissue culture flasks in a humidified atmosphere containing 5% CO_2_ with temperature set at 37 °C. By using 1% trypsin-ethylene-diamine tetra-acetic acid (trypsin-EDTA) solution, cells were regularly detached from the surface of flasks. Recombinant human VEGF (VEGF165) was bought from R&D systems (Minneapolis, MN, USA). The following antibodies: phospho-VEGFR2 (Tyr1175) (19A10) (catalog No: #2478S), VEGFR2 (55B11) (catalog No: #2479S), phospho-eNOS (Ser1177) (catalog No: #9571L), eNOS (catalog No: #9572S), phospho-p44/42 MAPK (Erk1/2) (Thr202/Tyr204) (catalog No: #9101S), p44/42 MAPK (Erk1/2) (catalog No: #9102S), phospho-Akt (Ser473) (catalog No: #9271S), Akt (catalog No: #9272S) and GAPDH (D16H11) (catalog No: #5174) were obtained from Cell Signaling Technology (Danvers, MA, USA). Semaxanib (SU5416) was bought from Sigma-Aldrich (St. Louis, MO, USA). Piceatannol was a gift from Testing Laboratory for Chinese Medicine of HKUST, and its purity was more than 98% as determined by HPLC-DAD. A stock solution of piceatannol with the concentration set at 100 mM was freshly dissolved in dimethyl sulfoxide (DMSO).

### 4.2. Cell Viability Assay

The 3-(4,5-dimethylthiazol-2-yl)-2,5-diphenyltetrazolium bromide (MTT) assay was, first of all, applied to investigate the effects of piceatannol on cultured HUVECs [29]. Briefly, HUVECs at a density of 5 × 10^3^/mL and colon cancer cells at a density of 1 × 10^4^/mL were separately plated into each well of a sterile 96-well plate. After incubated for 24 h, the medium was changed with 100 μL of fresh medium containing VEGF (10 ng/mL) or different concentrations of piceatannol, and the drug treatment lasted for 48 h. The cell viability was determined as previously reported [29]. The release of LDH was detected with application of a cytotoxicity detection kit plus (LDH) (Roche Diagnostics, Indianapolis, IN, USA) following the manufacturer’s instruction. To quantity the content of LDH, the followed formula was used: Cytotoxicity (%) = (experimental value − low control)/(high control − low control) × 100.

### 4.3. Endothelial Cell Migration Assay

A wound-healing assay was used to determine the migratory ability of endothelial cells [29]. Briefly, HUEVCs, at a density of 2 × 10^5^ cells/well, were seeded into each well of a sterile 12-well plate. After cells incubated for 24 h, with application of a sterile 200 μL plastic pipette tip, a single and straight wound was manually made at the center of cell monolayer. To remove scratched cells, prewarmed PBS was used to wash cell. Images were taken under a phase-contrast microscope (A_t0_). After cells incubated with complete medium containing VEGF (10 ng/mL) or Avastin/piceatannol for 8 h, four wound fields were chosen randomly and photographed (A_t8_). By using Tscratch software (CSE Lab, Switzerland), the scratched area of each group before and after drug treatments was determined and based on the below formula: Recovery (%) = A_t0_ − A_t8_/A_t0_ × 100%, the recovery percentage of cells was calculated.

### 4.4. Endothelial Cell Invasion Assay

The cell invasion ability of endothelial cells was determined by using a Transwell Boyden chamber (8-μm pore; Corning Inc., Lowell, MA, USA). The chamber was precoated with Matrigel and was allowed to polymerize for 1 h at 37 °C. Next, 2 × 10^4^ cells in 100 μL serum-free medium with or without piceatannol and 10 ng/mL VEGF, were plated on the upper compartment. At the same time, the lower chambers were supplied with 500 μL complete medium. The drug treatment lasted for 24 h. After drug incubation, prewarmed PBS was used to rinse off the endothelial cells those failed to attach and grow; while cells succeeded invading into lower surface were first, dehydrated with ethanol and then stained with 0.01% crystal violet. Finally, pictures of stained cells in the lower surface were taken under a phase-contrast microscope and were further quantified by counting cells manually. The invasion ratio was demonstrated as percent of the control.

### 4.5. Endothelial Cell Tube Formation Assay

Each well of a sterile 24-well plate was first, precoated with Matrigel and allowed to polymerize for 1 h at 37 °C, as described [29,54]. Then, endothelial cells containing VEGF (10 ng/mL) with or without piceatannol at different concentrations were seeded onto Matrigel-precoated each well of plates. The cell density was set at 1 × 10^5^ cells/well. After cells treated with drugs for 8 h at 37 °C, tube-like networks were formed, and pictures were randomly taken under a phase-contrast microscope. Images were further quantized by identifying the number of the branching points. Four fields in each well were randomly determined and counted manually.

### 4.6. Zebrafish Angiogenesis Assay

The in vivo anti-angiogenic effects of piceatannol were investigated based on wild-type zebrafish model. Zebrafish was fed in a system provided with regular aeration and flow water, maintained at a cycle of 10 h:14 h of dark/light. Temperature was set at 28.5 °C and the humidity was maintained at 50 ± 10%. The experimental conduction and zebrafish maintenance were conducted followed the regulations of Animal and Plant Care Facility with approval of the Animal Ethics Committee of HKUST (Cap. 340). Based on natural spawning of mating matured fishes, embryos were obtained. The matured fishes were at least 6 months old. After 24 h, each embryo was dechlorinated manually and put into each well of 12-well plate at a density of 8–10 embryos per well. Embryos were then incubated with egg water containing VEGF with or without different concentrations of piceatannol at 28.5 °C. Embryos fed with egg water served as control. After drug treated for 48 h, viability and morphologic characteristics of embryos were reviewed under a phase-contrast microscope.

### 4.7. Alkaline Phosphatase-based Vascular Staining

On the 3rd day of embryo development, embryos were fixed by immersing in paraformaldehyde (4%) for 20 h at 4 °C. After fixation, fish embryos were rinsed by PBS containing 0.1% Tween-20 (PBST) solution for 5 min and went through dehydration by using 50% methanol and methanol. Then, embryos immersed in methanol were stored at −20 °C refrigerator for 2 h. After dehydration, embryos were rinsed by PBST solution for four times, 5 min each. To stain the vessels, embryos were first, kept in buffer 9.5 T for half an hour at room temperature. The buffer 9.5 T was prepared by 1-M Tris-9.5, 1-M MgCl_2_, deionized water and 20% Tween-20. Then embryos were directly stained by freshly prepared nitro-blue tetrazolium/5-bromo-4-chloro-3-indolyl-phosphate (NBT/BCIP) (Cell Signaling Technology, Danvers, MA, USA). The staining reaction was taken under dark at room temperature. After 30 min, PBST solution was used to stop the staining reaction. The stained embryos were then washed by PBST solution for three times, 5 min each, to remove left NBT/BCIP. Finally, images representing subintestinal vessels of embryos were taken with application of a stereomicroscope (Nikon AZ100, Nikon, Tokyo, Japan) equipped with a digital camera (Olympus DP71, Tokyo, Japan). The branch number and area of subintestinal vessels were analyzed by Image J software (v2.1.4.7, National Institutes of Health, Bethesda, MD, USA). The experiment was independently conducted in triplicate. Same numbers of fish embryos were used for each group.

### 4.8. Molecular Docking

The structure of VEGF was downloaded from Protein Data Bank (PDB); while structures of compounds, including piceatannol (PubChem: 667639), pinosylvin (PubChem: 5280457), 3,4′,5-trimethoxy-*trans*-stilbene (PubChem: 5388063), were from NCBI-PubChem database. Before molecular docking analysis, the structures of compounds were transferred into MOL2 mode with application of Chemoffice 2014 software (Cambridge Soft, Cambridge, MA, USA). Then, the docking analysis was conducted based on compound—VEGF protein model. With application of AutoGrid program, affinity (grid) maps of 40 × 40 × 40 Å grid points, corresponding to x, y and z and 0.375 Å spacing, were clearly shown. The box center was optimized as followed: x: 0.38 Å, y: −2.98 Å and z: 20.51 Å [55]. Having these parameters determined, the binding mode was suitable for as many binding interactions as possible. The energies could also be roughly estimated [56]. By using vina software, the binding activities of piceatannol and VEGF were analyzed, and the values representing binding affinity were determined.

### 4.9. Immunoprecipitation Assay

One hundred µL of piceatannol solution with concentration set at 0.5 µM was first, reacted with VEGF or biotinylated VEGF at 4 °C for 60 min, and 100 µL of PureProteome streptavidin magnetic beads were added separately to incubate together with protein–compounds complexed solutions. The binding reaction lasted for 24 h at 4 °C. The magnetic stand acted to accumulate the beads at the bottom of magnet. Without touching the beads, the supernatant was collected, and the VEGF/biotin-labeled VEGF complex was separately washed by PBS solution for three times, 5 min each. Then, the VEGF/biotin-labeled VEGF complex was precipitated by using acetonitrile. To determine amount of piceatannol in supernatant, UPLC instrument was used, and the supernatant was analyzed with an Agilent, Grace VisionHT C18 column (4.6 × 250 mm, 5 μm). Acetonitrile (Solvent A) and 0.2% diluted aqueous formic acid (Solvent B) were used as mobile phases, and the gradient elution was determined as followed: Solvent A was gradually increased from 2% to 18% by 18 min, from 18% to 45% by 70 min and finally from 45% to 90% by 96 min. The column temperature was set at 30 °C, and the wavelength was optimized at 254 nm, accompanied with a whole spectral scanning at a range of 190 to 400 nm.

### 4.10. Western Blot Analysis

The expressions of phosphorylated molecules, e.g., VEGFR2 (Tyr1175), Akt (S473) and p44/42 MAPK (Erk1/2) (Thr202/Tyr204), were determined by western blotting analysis. Before drug treatment, endothelial cells were incubated in the medium without serum for 60 min. After drug incubation, samples were collected by using freshly prepared low-salt lysis buffer. The lysis buffer was composed of 10% glycerol, 2% SDS, 125-mM Tris-HCl and 200-mM 2-mercaptoethanol. The pH value of lysis buffer was 6.8 and then the collected samples were boiled with temperature set at 95 °C for three times, 5 min each. During the boiling interval of each time, the samples were vortexed. The boiled protein extracts were subjected to be separated into different sizes of protein based on a 7% or 8% acrylamide gel—and by applying the electrophoresis method—the gels were transferred onto nitrocellulose membranes. The transferring reaction was taken for 15 h at 40 V voltage in a 4 °C room. Then, membrane standing for targeted size of proteins was acquired and subjected to be blocked by using 5% milk solution. The milk solution was diluted in a Tris-buffered saline containing 0.1% Tween-20 solution (TBST). The blocking reaction was taken at room temperature for 60 min and after blocking reaction finished, membrane was incubated with various kinds of primary antibody solutions for at least 15 h at 4 °C. Based on the instruction, antibodies were diluted at a 1:1000. After incubated with primary antibodies, blots were rinsed by using TBST solution and reacted with horseradish peroxidase-conjugated secondary anti-rabbit antibody. The applied secondary antibodies were diluted at a 1:2000. After incubated with secondary antibody for 120 min, membranes were rinsed with TBST solution for four times, 5 min each. By using ECL (Invitrogen), the reactive bands were visualized and taken pictures under Chemidoc Imaging System (Bio-Rad; Hercules, CA, USA). The intensities of immune-reactive bands were determined. The band intensities from the control group and different drug-treated groups, run on the same piece of gel and performed under standardized ECL conditions, were analyzed and further measured on specialized software for image analysis, on a basis of a calibration plot from a parallel gel with one of the samples diluted at a series of ratios. For the quantification of western blot in phosphorylation, the band at 10 min from each group was in comparison with the control at 0 min.

### 4.11. Measurement of Reactive Oxygen Species

For the determination of ROS formed, 2′,7′-dichlorofluorescein diacetate (DCFH-DA) was applied. After treated by piceatannol and VEGF (10 ng/mL) for 48 h, endothelial cells were incubated with DCFH-DA at 37 °C for 30 min. Here, the concentration of DCFH-DA used was 100 μM. Then cells were washed by prewarmed PBS for three times, 5 min each, and pictures were taken under a laser confocal fluorescent microscopy. For image analysis, total green fluorescence intensity of each group was measured with relevant software.

### 4.12. Transwell^®^ Motility Assay

HT-29 colon cancer cells were seeded onto the polycarbonate filter of upper chamber of Nunc™ cell culture inserts in carrier plate systems in free medium with 10 ng/mL VEGF with or without Avastin or a series of concentrations of piceatannol, in parallel, the culture medium supplied with 10% FBS containing 10 ng/mL VEGF in the presence or absence of piceatannol was added into lower chamber. After incubated with drugs for 24 h, cells left on the top side of polycarbonate Transwell^®^ filters were carefully removed by using cotton swabs. Cells migrated into opposite side were subjected to be on fixation for 15 min at room temperature by using 4% formaldehyde in 1× PBS. The nuclei of cells were stained by DAPI, and ten images were randomly captured for each well by using a fluorescence microscopy (DMIRE2, Leica, Wetzlar, Germany) equipped with Leica confocal software (Version 2.61), 10× objective. The wavelengths for excitation and emission were separately set at 358 nm and 461 nm. Total cell number of 10 images was counted for each well.

### 4.13. Other Assays

Protein concentrations were measured by the Bradford method with a kit from Bio-Rad. Statistical analysis was performed using one-way analysis of variance (ANOVA) followed by a Bonferroni multiple comparisons test using the SPSS 16.0 software. Data were demonstrated as the mean ± standard error of the mean (SEM). The statistical significance was set at *p* < 0.05.

## 5. Conclusions

This study explores anti-angiogenic and anticancer effects of piceatannol in VEGF-induced endothelial cells and human colon cancer cells, and its related signaling mechanisms are further identified. Piceatannol could have the potentials to be used as an anti-angiogenic drug. Such activities of piceatannol, as a natural compound, is different from common-used anti-angiogenic medicines and which could be further applied for prevention and treatment of angiogenesis-related diseases, including cancers. The present study demonstrating basic pharmacological information may give supports to the development of anti-angiogenic reagent deriving from natural products.

## Supplementary Materials

The following are available online at https://www.mdpi.com/1420-3049/25/17/3769/s1, Figure S1: VEGF promotes endothelial cell proliferation in a dose dependent manner, Figure S2: Piceatannol exerts inhibitory effects in VEGF triggered cell proliferation and piceatannol alone does not affect endothelial cell morphology, Figure S3: Piceatannol analogues bind with VEGF, Figure S4: Piceatannol suppresses VEGF induced cancer cell proliferation, Figure S5: Effects on expressions of P-VEGFR2 and VEGFR2, Figure S6: Effects on expressions of P-VEGFR1 and VEGFR1, Figure S7: Effects on expressions of P-Akt and Akt, P-Erk and Erk, Figure S8: Effects on expressions of specific molecules.

## Abbreviations

VEGFs	vascular endothelial growth factors
HUVECs	human umbilical vein endothelial cells
VEGF	vascular endothelial growth factor
VEGFR1	VEGF receptor 1
VEGFR2	VEGF receptor 2
VEGFR3	VEGF receptor 3
Erk	extracellular signal-regulated kinase
GAPDH	glyceraldehyde 3-phosphate dehydrogenase
