# Supplementary material for: Piceatannol, a Natural Analog of Resveratrol, Exerts Anti-angiogenic Efficiencies by Blockage of Vascular Endothelial Growth Factor Binding to Its Receptor
Source: Molecules. 2020 Aug 19;25(17):3769. doi: 10.3390/molecules25173769 (PMC7504081; doi:10.3390/molecules25173769)
Supplement: Supplementary file 1 [file molecules-25-03769-s001.pdf]

Piceatannol, a natural analogue of resveratrol. exerts anti-angiogenic efficiencies by blockage of vascular endothelial growth factor binding to its receptor

Wei-Hui Hu[1,2,3], Diana Kun Dai[2,3], Brody Zhong-Yu Zheng[2,3], Ran Duan[2,3], Tina Ting-Xia Dong[2,3], Qi-Wei Qin[1], Karl Wah-Keung Tsim*[,2,3]

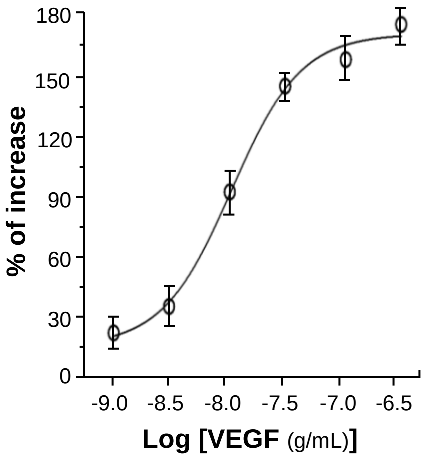

Supplementary Figure 1. VEGF promotes endothelial cell proliferation in a dose-dependent manner.

HUVECs were seeded into each well of a 96-well plate with cell density set at 5,000 cells/well, and treated by a series of concentrations of VEGF. The viability of endothelial cells was potentiated by applied VEGF in a dose-dependent manner. Data are showed as Mean ± SEM of the percentage of control or change in comparison to control group, where $n = 4$; $p < 0.05$ (*); $p < 0.01$ (**); $p < 0.001$ (***) vs VEGF-treated group.

Supplementary Fig. 1

Hu et al 2020

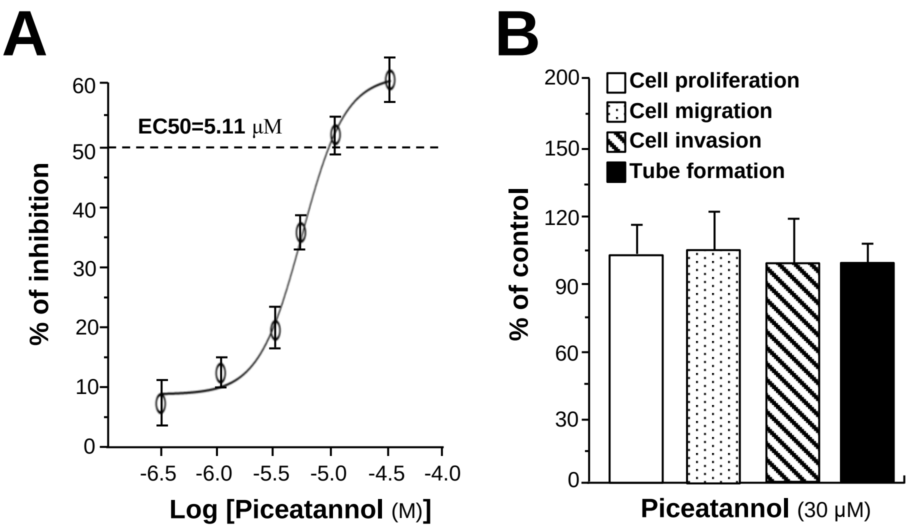

Supplementary Figure 2. Piceatannol exerts inhibitory effects in VEGF-triggered cell proliferation and piceatannol alone does not affect endothelial cell morphology. **(A)** HUVECs were seeded into each well of a 96-well plate with cell density set at 5,000 cells/well, and treated by VEGF (10 ng/mL) with a series of concentrations of piceatannol. The viability of endothelial cells potentiated by VEGF was suppressed by piceatannol in a dose-dependent manner, and EC50 of piceatannol was 5.11 μM. **(B)** 20×10^4 HUVECs were plated into each well of a 12-well plate. The cells were incubated with piceatannol (30 μM) for 48 hours. The identification of cell morphology was described as in Fig. 2. Data are showed as Mean ± SEM of the percentage of control or change in comparison to control group, where $n = 4$; $p < 0.05$ (*); $p < 0.01$ (**); $p < 0.001$ (***) vs VEGF-treated group.

Supplementary Fig. 2

Hu et al 2020

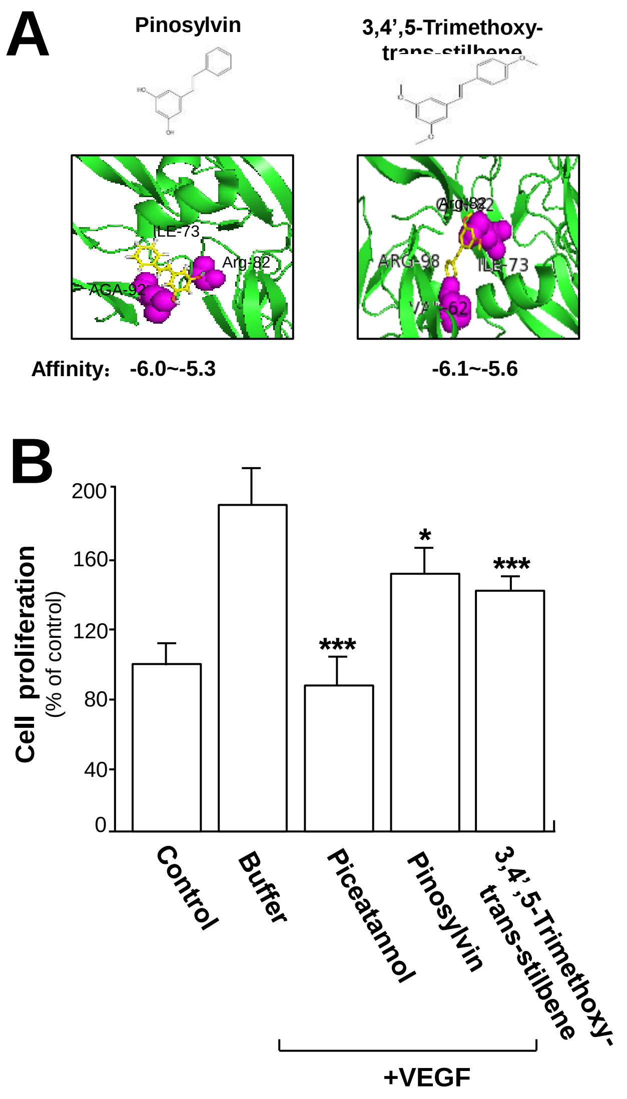

Supplementary Figure 3. Piceatannol analogues bind with VEGF.

(A) The binding interactions of piceatannol analogues with VEGF were analyzed based on an AutoDock software. VEGF: green; pinosylvin and 3,4',5-trimethoxy-trans-stilbene: sticks, carbon colour: yellow, oxygen: red, hydrogen: silver; the predicable binding site: purple. The values representing the binding affinity of piceatannol analogues with VEGF were demonstrated. **(B)** HUVECs were plated into each well of a 96-well plate with cell density set at 5,000 cells/well, incubated with piceatannol or its analogues for 48 hours with or without VEGF (10 ng/mL). Here, the applied concentration of piceatannol and analogues (pinosylvin and 3,4',5-trimethoxy-trans-stilbene) was 30 μM. Data are demonstrated as Mean ± SEM of the percentage of change as compared with control group (no treatment), where $n = 4$; $p < 0.05$ (*); $p < 0.001$ (***) vs VEGF-treated group.

Supplementary Fig. 3

Hu et al 2020

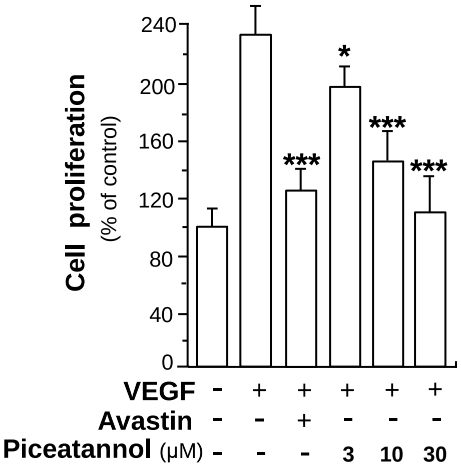

Supplementary Figure 4. Piceatannol suppresses VEGF-induced cancer cell proliferation.

Cultured colon cancer cells were incubated with a series of concentrations of piceatannol for 48 hours, and MTT assay was performed. Cancer cells seeded into each well of a 96-well plate with cell density set at 10×10^3 cells/well, were treated with piceatannol for 48 hours in the presence or absence of VEGF (10 ng/mL). Data are in percentage of control group, as Mean $\pm$ SEM, where $n = 4$; $p < 0.05$ (*); $p < 0.001$ (***) vs VEGF-treated group.

Supplemental data for **Fig. 4A**. Effects on expressions of P-VEGFR2 and VEGFR2
Representative unprocessed western blots of control and drugs treated groups as shown in Fig. 4A. Red box showed cropped area included in Fig. 4A.

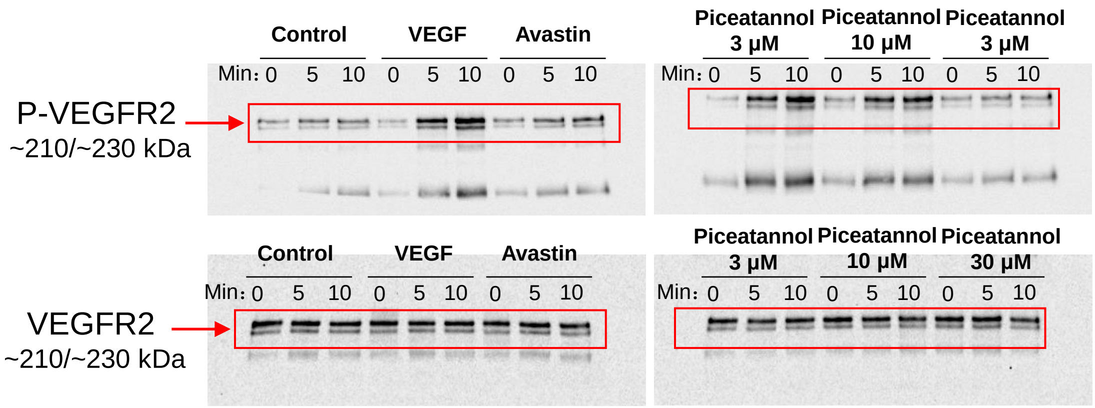

Supplemental data for **Fig. 4B**. Effects on expressions of P-VEGFR2 and VEGFR2
Representative unprocessed western blots of control and drugs treated groups as shown in Fig. 4B. Red box showed cropped area included in Fig. 4B.

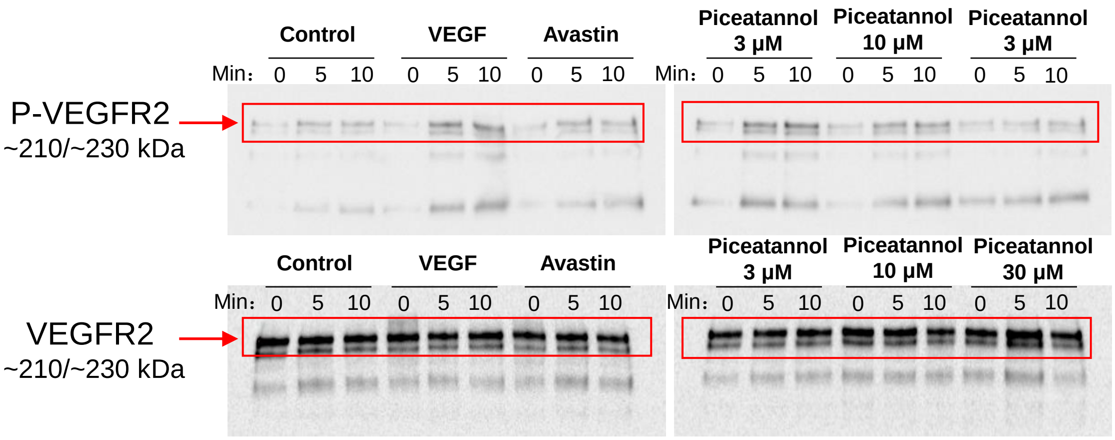

Supplementary Fig. 5

Hu et al 2020

Supplemental data for **Fig. 5**. Effects on expressions of P-VEGFR1 and VEGFR1
Representative unprocessed western blots of control and drugs treated groups as shown in Fig. 5. Red box showed cropped area included in Fig. 5.

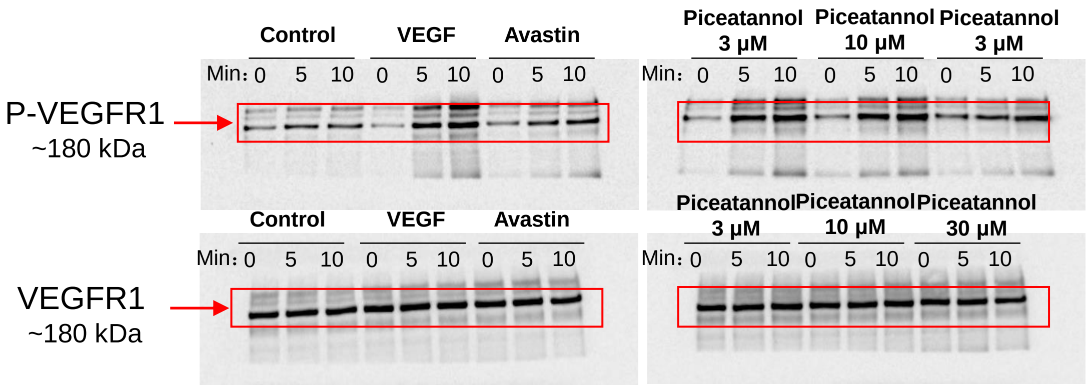

Supplementary Fig. 6

Hu et al 2020

Supplemental data for **Fig. 6A**. Effects on expressions of P-Akt and Akt
Representative unprocessed western blots of control and drugs treated groups as shown in Fig. 6A. Red box showed cropped area included in Fig. 6A.

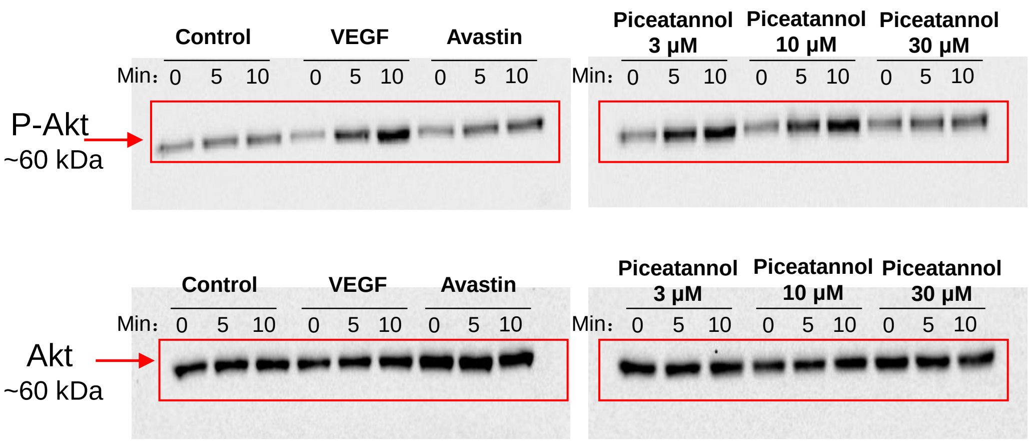

Supplemental data for **Fig. 6B**. Effects on expressions of P-Erk and Erk
Representative unprocessed western blots of control and drugs treated groups as shown in Fig. 6B. Red box showed cropped area included in Fig. 6B.

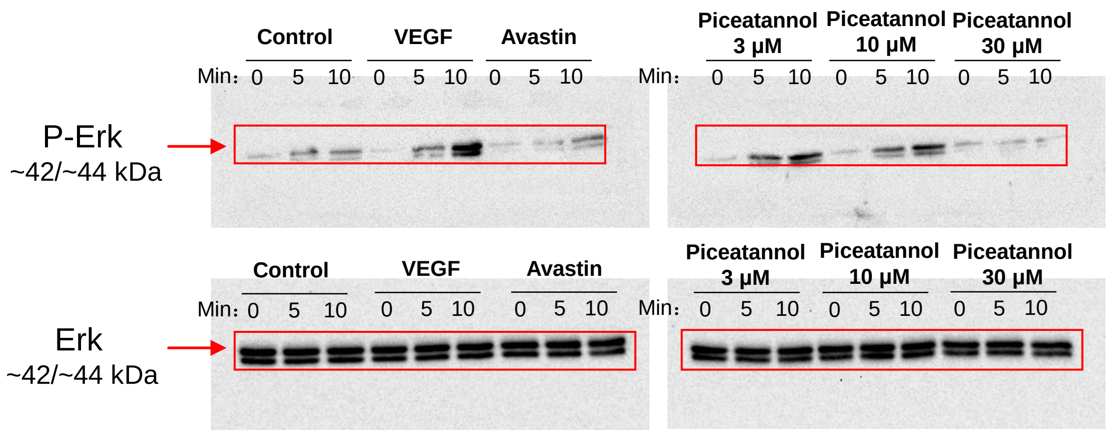

Supplementary Fig. 7

Hu et al 2020

Supplemental data for **Fig. 8B**. Effects on expressions of specific molecules

Representative unprocessed western blots of control and drugs treated groups as shown in Fig. 8B. Red box showed cropped area included in Fig. 8B.

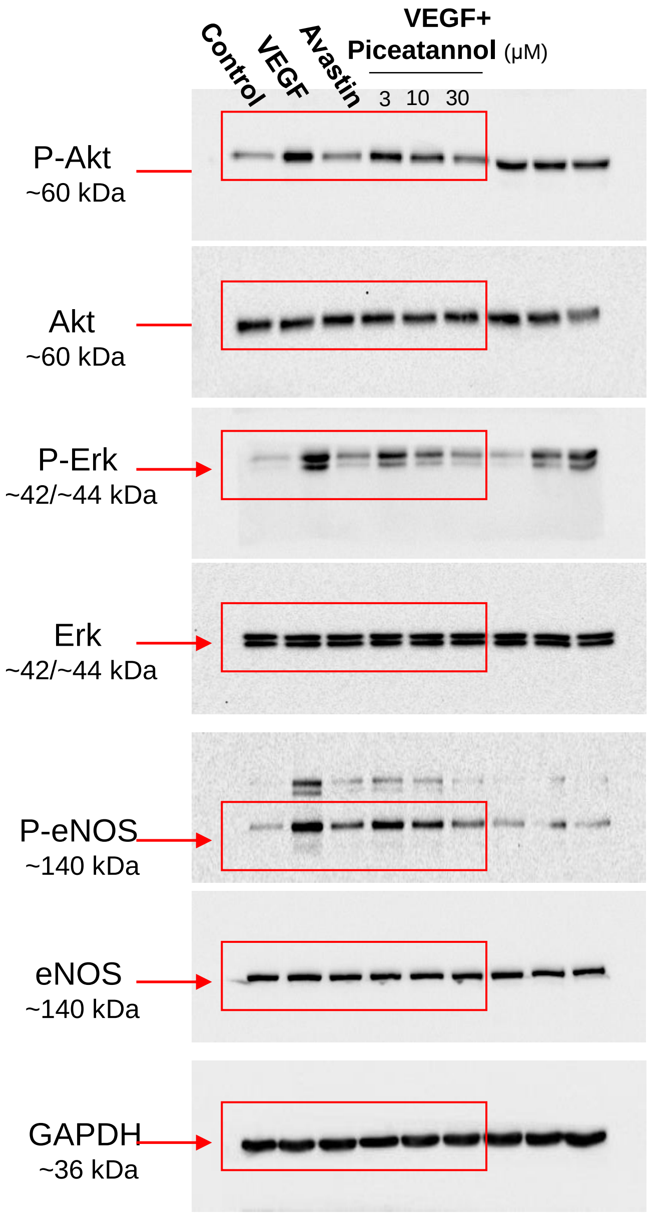

Supplementary Fig. 8

Hu et al 2020
